# Cholera Toxin Subunit B as Adjuvant––An Accelerator in Protective Immunity and a Break in Autoimmunity

**DOI:** 10.3390/vaccines3030579

**Published:** 2015-07-24

**Authors:** Thomas Stratmann

**Affiliations:** Department of Physiology and Immunology, Faculty of Biology, University of Barcelona, Av. Diagonal 643, 3rd floor, Barcelona 08028, Spain; E-Mail: thomas.stratmann@ub.edu; Tel.: +34-934021533

**Keywords:** Cholera toxin subunit B (CTB), adjuvant, expression, oral tolerance, immunity, autoimmunity

## Abstract

Cholera toxin subunit B (CTB) is the nontoxic portion of cholera toxin. Its affinity to the monosialotetrahexosylganglioside (GM1) that is broadly distributed in a variety of cell types including epithelial cells of the gut and antigen presenting cells, macrophages, dendritic cells, and B cells, allows its optimal access to the immune system. CTB can easily be expressed on its own in a variety of organisms, and several approaches can be used to couple it to antigens, either by genetic fusion or by chemical manipulation, leading to strongly enhanced immune responses to the antigens. In autoimmune diseases, CTB has the capacity to evoke regulatory responses and to thereby dampen autoimmune responses, in several but not all animal models. It remains to be seen whether the latter approach translates to success in the clinic, however, the versatility of CTB to manipulate immune responses in either direction makes this protein a promising adjuvant for vaccine development.

## 1. Introduction

Cholera toxin is the soluble toxin secreted by the Gram negative bacteria *Vibrio cholerae*. The toxin is composed of two proteins, the subunit A (CTA) that exists as a monomer in the complex, and the subunit B (CTB) that forms a pentamer. The dual protein nature of cholera toxin was initially recognized by Lönnroth and Holmgren in 1973 [1], and structural studies of the closely related heat-labile enterotoxin from *Escherichia coli* [2] and subsequently of cholera toxin and CTB confirmed these initial findings [3,4]. CTB forms a ring-like structure composed of five CTB monomers. Each monomer interacts with two neighboring molecules through hydrogen bonds and salt bridges without being covalently linked to each other. The center of this pentameric formation adopts a tunnel-like structure, the wall of which is formed by five alpha helices (α2), each one belonging to a different monomer. CTA has a globular structure with a protruding C-terminal alpha helical extension. This extension inserts into the tunnel formed by the CTB pentamer that accepts only a single CTA molecule, leading the final AB_5_ hexameric structure of cholera toxin [5]. CTA is generated as a single protein chain, however, due to a proteolytic cleavage CTA is split into two subunits, CTA1 and CTA2, that remain combined in the hexamer until the complex has entered the host cells and reached the endoplasmatic reticulum. CTA1 corresponds to the globular portion of CTA, while CTA2 forms the protruding alpha helix that inserts into the tunnel by the pentamer. The toxic portion of cholera toxin is CTA, and more specifically, CTA1. After cell entry, CTA1 eventually reaches the endoplasmatic reticulum and next the cytosol [6] where it interacts with adenylate cyclase, leading to its activation. Adenylate cyclase catalyzes the conversion of ATP to 3',5'-cyclic AMP which in turn is responsible for the activation of cystic fibrosis transmembrane conductance regulator (CFTR) channel [7]. This leads to a salt imbalance of the epithelial cells in the gut characteristic for enterotoxic poisoning. Epithelial cells under these conditions increase the extrusion of chloride ions, and simultaneously the uptake of sodium is prevented. This leads to a fatal loss of water by the intestine.

The cellular uptake of cholera toxin is regulated by the recognition of its receptor, the monosialotetrahexosylganglioside (GM1a, Galβ3GalNAcβ4(Neu5Acα3)Galβ4GlcCer; for nomenclature and structure please view [8]). The identity of GM1 as a receptor for cholera toxin was elaborated by Holmgren and colleagues in 1973 [9]. CTB interacts with GM1 through its pentasaccharide moiety. Each CTB monomer interacts primarily with one pentasaccharide, however, each pentasaccharide also extends its contacts to an adjacent CTB molecule. This explains why CTB needs to adopt a pentameric formation to gain maximal function. The optimal sugar binding of two B subunits is only then combined with the additional avidity obtained by simultaneous interaction with more than one GM1 ganglioside. The pentasaccharide-CTB interaction further enhances the interaction between B subunits within the pentamer [10].

The fact that CTB is non-toxic has made this molecule an excellent delivery vehicle, given the cellular distribution of its receptor. GM1 is found on a broad variety of cell types including neurons [11]. Pertinent to this review is the presence of this ganglioside on the surface of the epithelial cells in the gut as this is a crucial entry point into the body when using CTB in oral vaccine formulations, and on antigen presenting cells. Apart from macrophages and dendritic cells [12], GM1 is also expressed by B cells in which upon CTB binding expression of MHC class II is increased [13]. This vastly increases antigen presentation of CTB and molecules coupled to it in the organism. In a homeostatic setting, typically only a very limited number of Ag-specific B cells exists in the total B cell repertoire that will efficiently capture Ag via the B cell receptor (BCR) recognizing the Ag and guide it towards the MHC class II loading compartment. Ag capture via CTB and GM1 bypasses this loophole, which therefore potentially converts the entire B cell compartment into potential antigen presenters, in addition to macrophages and dendritic cells.

## 2. CTB Expression in Bacteria and Yeast

The CTB monomer is composed of 124 amino acids including the leader peptide of 21 residues. The mature form contains 103 amino acids and has a molecular weight of 11.6 KD [14]. CTA and CTB, after synthesis in the cytoplasm, are guided to the periplasm. Here, CTB assembles to form the pentamer which is either secreted separately or may bind to CTA to form the holotoxin AB5 which next is secreted [15]. Its relatively small size and its close similarity to the enterotoxin from *E. coli* facilitates its recombinant expression in this host. In *E. coli*, recombinant overexpression of CTB is generally carried out omitting the leader peptide in the expression constructs, leading to the formation of inclusion bodies. However, the protein can be solubilized in urea and readily refolds under proper conditions [16]. The protein is well expressed, with typical yields of purified protein exceeding 100 mg/L of bacterial culture volume in our laboratory. Proper refolding and formation of the pentameric structure needs to be verified e.g., by size exclusion chromatography and by SDS-PAGE under native conditions in which the pentamer maintains its conformation [17]. The functionality of the pentamer can be tested by its binding capacity to immobilized D-galactose [18]. CTB has further been expressed in *Lactobacillus* and its immunogenicity verified by challenging mice intranasally with purified CTB [19].

Miyata and colleagues reported the expression of CTB in methylotrophic yeast (*Pichia pastoris*) [20] (Table 1). Compared to bacterial expression, the expression in yeast has the advantage of being conveniently similar to bacterial culture, yet without the generation of endotoxins. Other advantages of yeast, to take into consideration when fusing CTB to proteins that need proper folding, are the disulfide bond formation and the glycosylation. Approximately 50 mg/L of culture can be obtained in this system. Another methylotrophic yeast species, *Hansenula polymorpha*, has been used to express CTB fused N-terminally to short epitopes of the foot-and-mouth disease virus. Up to 100 mg of the fusion protein was obtained per liter of culture volume in this system [21].

**Table 1:** Expression systems of CTB and CTB-fusion proteins.

Expression System	CTB Fused to	Reported Yields	Ref.
*E. coli* BL21	Fimbria 2 (*Bortedella* *pertussis*), C-term.	Not specified	[22]
*E.* *coli* BL21	Adhesin from Streptococcus, fused to CTA2, CTB coexpressed but unfused	3.5 mg/L of culture	[23]
*E. coli* KS476	Albumin binding region (BB) of Streptococcus protein G N-terminal, C-terminal and both, N- and C-terminal	6–12 mg/L of culture	[24]
*E.* *coli* BL21 (DE3)	Cedar pollen Ag cry j 1 and cry j 2, C-terminal	120 mg/L culture volume	[25]
*Lactobacillus casei Lactobacillus reuteri*	Hexahistidine tag or unfused	0.05 mg/L unfused, 1 mg/L when fused to histidine tag	[19]
*Lactobacillus* *casei*	YVAD tetrapeptide (caspase-1 inhibitor), C-terminal	1 mg/L culture volume	[26]
Yeast (*Pichia* *pastoris*)	CTB stabilized by fusion to five stranded α-helical coiled-coil domain of cartilage oligomeric matrix protein(COMP)	Not specified	[27]
Silkworm (*Bombyx mori*)	amyloid-β peptide(Aβ42), C-term	0.5 µg/g silkworm pupae	[28]
Silkworm (*Bombyx mori*)	Insulin-GFP	0.58 mg/mL of hemolymph, 0.23 mg/larva	[29]
Tobacco (*Nicotiana benthamiana*)	unfused	>1 g/kg of fresh leaves	[30]
Tobacco (*Nicotiana* *benthamiana*)	HIV membrane proximal (ectodomain) region of gp41, C-term	1–2 mg purified protein/kg of fresh leaves	[31]
Tobacco (*Nicotianatabacum* var. *Petit* *havana*)	unfused	4.1% of total soluble leaf protein	[32]
Tobacco (*Nicotiana* *tabacum* var. *Petit* *havana*), chloroplast	Mycobacterium Ag ESAT-6 (6 kDa early secretory antigenic target) and Mtb72F (a fusion polyprotein from two TB antigens, Mtb32 and Mtb39)	0.95 mg/g fresh leaf (CTB-ESAT-6), 1.1% of total soluble protein (CTB-Mtb72F)	[33]
Tobacco (*Nicotiana* *tabacum*)	N-terminal fused to p201 epitope from ApoB100 and CETP 461–476, C-terminal	10 mg/kg of fresh plant leaf	[34]
Tobacco	MBP,C-terminal	2% of total leaf protein	[35]
Rice (*Oryza* *sativa* japonica, *Nipponbare*)	Unfused, Asn to Gln substitution at 4th glycosylation position	2.3 mg/g of seed CTB unglycosylated	[36]

Apo: Apolipoprotein; CETP: cholesteryl ester transfer protein; CETP: cholesteryl ester transfer protein; MBP: myelin basic protein.

## 3. CTB Expression in Plants

CTB has been expressed successfully in various plant systems, for example in tobacco (*Nicotiana benthamiana*), where the protein, unlike the bacterially expressed form, gets glycosylated, presumably at position Asn25, which does not affect the pentamer formation [31]. Its binding capacity to GM1 was found to be decreased approximately five-fold in an ELISA assay in comparison to bacterially expressed CTB, however, in a hemagglutination assay using GM1 coated sheep red blood cells both proteins were indistinguishable, probably as this assay is less sensitive [31]. In this particular case, CTB was fused to the membrane proximal (ectodomain) region of gp41 (MPR649–684), and made up 0.14% of the total soluble protein, or approximately 20 mg per kg of fresh leaf material. The protein was immunogenic, and although no direct side-by-side comparison with the bacterial version of the same construct in the study was carried out, the authors noted that three doses were sufficient to raise a vaginal IgA towards the protein, something they did not achieve in previous studies using bacterially expressed protein [37]. The authors speculated that this might be due to the interaction between the high mannose glycans of the glycosylated CTB and the mannose receptors on the surface of APCs. The same group reanalyzed in a subsequent study unfused glycosylated CTB *versus* bacterial CTB and found that both proteins bound almost equally well to GM1 in a low nanomolar range [38]. In that case a side-by-side comparison of immunogenicity between both proteins was conducted. No statistically significant differences were detected in fecal IgA, serum IgG or the composition of IgG subtypes. Preventing the N-terminal glycosylation led to necrosis in the plant tissue and a reduction in production yields. N-glycosylation reduced ER stress and improved proper folding of the protein [38]. CTB expression in this system fused to epitopes of proteins relevant to arteriosclerosis, namely the apolipoprotein ApoB100 that has been implicated in inflammation and depositions, leading to chronic inflammation of the arteries, as well as a second protein implicated in this process, the cholesteryl ester transfer protein (CETP), has been achieved. The fusion proteins were capable to generate Abs *in vivo* against the epitopes of interest in BALB/c mice [34]. Thus, the integrity of the epitopes was maintained in the plant. Another group, however, reported that elimination of *N*-glycosylation of CTB, expressed by its own, led to an improved expression yield in *Nicotiana*. The authors showed that this nonglycosylated CTB had similar properties compared to bacterial CTB, and when orally administered to mice could generate protective anti-toxin Abs [30].

A different approach of subcellular localization in *Nicotiana*, *i.e.*, the expression in chloroplasts, has been chosen by Lakshmi *et al.* [33]. This was achieved by cloning CTB fused to *Mycobacterium tuberculosis* derived epitopes, *i.e.*, the 6 KD early secretory antigenic target (ESAT-6) and Mtb72F (a protein resulting from the fusion of two TB antigens, Mtb32 and Mtb39), downstream of the psbA promoter that normally regulates expression of the protein D1, a component of the Photosystem II. CTB in chloroplasts formed functional GM1 binding pentamers, however, whether or not the vaccine lead to a protective immune response was not investigated in this study. Chloroplast expression of CTB has also been achieved by the generation of targeting vectors, leading to insertion of CTB coding DNA into chloroplast DNA. The expressed protein was found to make up 4% of the total plant protein, and functional pentamers accumulated in the chloroplasts. The plant-expressed pentamers bound to GM1 in an ELISA format as did bacterially expressed CTB; however, since total plant extract was used rather than purified CTB, no detailed comparative conclusions on the GM1 binding capacity could be drawn [32].

Expression of CTB has also been successful in rice to serve as cholera vaccine [39,40,41] that was protective in mice and macaques when administered orally [36,41]. The intent to reduce by RNAi the expression of rice allergens that could lead to adverse effects in humans had the beneficial effect that in these plants CTB expression was improved several-fold. This was accompanied with a change of intracellular localization of CTB. Whereas in plants without RNAi suppression CTB was detected in the cytoplasm and in protein bodies II, in the presence of RNAi suppression CTB was found in the cytoplasm, the plasma membrane and the cell wall [42].

## 4. CTB Expression in Silk Worms

CTB fusion proteins have further been successfully produced in silkworms. In one study, CTB fused to the 42 amino acid isoform of the amyloid-β peptide Aβ42 in this system was successfully used in a mouse model for Alzheimer’s disease to reduce Aβ deposition in the brain [28]. A fusion product of CTB and insulin in the same system has been claimed to reduce insulitis and diabetes onset when administered orally in a relevant type 1 diabetes model, the nonobese diabetic (NOD) mouse [43,44]. The same results were obtained with a GFP-tagged CTB-insulin fusion protein (CTB-INS-GFP) generated to analyze binding of the protein to the intestinal wall [29].

## 5. Formulation of CTB as Adjuvant

When using CTB as adjuvant to direct the protein of interest to the desired cells, several approaches can be taken (Table 1 and Table 2). A common form is to fuse CTB recombinantly to the antigen of interest. A systemic analysis has evaluated the C-terminal *versus* the N-terminal fusion to CTB [24]. For this study, a 25 KD fusion partner, the serum albumin binding region (BB) of protein G from *Streptococcus*, was fused either N-terminally, C-terminally, or C and N-terminally simultaneously. While CTB as fusion partner was found to be quite permissive, *i.e.*, all three proteins were expressed and formed pentamers, of the three fusion proteins, the C-terminal fusion led to the best pentamer formation and showed highest affinity to GM1. In case that either CTB does not fold properly, the pentamer does not form or the binding capacity to GM1 is affected, a different approach can be used by replacing CTA1 with the Ag and fusing it N-terminally to CTA2; if this construct is coexpressed together with wild type CTB [23], a CTB pentamer will form, into which CTA2 will insert via the tunnel formed by CTB, leaving the Ag protruding out of the ring structure. This construct leaves the CTB moiety without any modification, and GM1 binding by the pentamer is thus optimal. However, this approach also decreases the molecular ratio of Ag to the CTB pentamer from 5:1 to 1:1, which would need to be taken into account when comparing vaccine dose of different fusion proteins, unless several Ag molecules or peptide epitopes are fused to each other in combination with CTA2. If the stability of CTB is an issue, e.g., when fused to larger proteins, the CTB pentamer can be further stabilized by engineering either cysteine bridges that interconnect the monomer between each other [45], or by the introduction of coiled coils at the C-terminus of recombinant CTB [46], leading to heat-stable formation of the pentamer in both cases.

Instead of recombinant fusion, CTB may be coadministered with Ag without being physically linked to each other, or it may be chemically coupled to the Ag. Furthermore, the protein can either be expressed and purified, or in the case of expression in plants, the purification may be omitted by generating an edible plant vaccine. Another way to circumvent protein purification is to include CTB into DNA vaccines.

Chemical coupling has the advantage that CTB on its own normally expresses very well while its expression with a fusion partner might decrease expression levels. In our hands for example, expression levels of CTB in *E. coli* were very high, reaching more than 100 mg of purified CTB per liter of culture volume. Fusion of even small peptides to the C-terminus led to decreased expression levels depending on the peptide, cutting down the total yield to a third in some cases (unpublished observation). Chemical coupling can be convenient when the response to synthetically produced peptides or commercially available proteins are to be analyzed. Chemical coupling can be achieved randomly by targeting amino groups [47]. A different approach to link antigen to CTB was used recently by Miyata and colleagues [20]. The group expressed CTB in yeast and coupled the antigen of interest, merozoite surface protein-1 from *Plasmodium yoelii*, via N-linked oligosaccharides of CTB. The authors reported that this conjugation led to a higher protection against a challenge with *Plasmodium* in mice compared to conjugation of the same protein to the CTB core protein.

**Table 2:** Adjuvant effect of CTB.

Antigen	Fused to CTB	(Animal) Model, Pathogen, Route	Analyzed Response	Ref.
OVA	conjugated	DO11.10OVATCR transgenic mice, i.g.	Induction of CD25 + Treg > 2-fold compared to OVA only,Treg have suppressor function	[47]
OVA	unfused or conjugated	DC *in* *vitro* exposed to OVA, CFA + CTB, OVA coupled to CTB and transfer to BALB/c mice, boost with OVA + CFA	IgG titers >3-fold increase, upregulation of CD80 and CD86 (CTB coupled to OVA *vs*. CTB + OVA or OVA only) Upregulation of IFN-γ (CTB coupled to OVA *vs.* OVA)	[48]
OVA	unfused	BALB/c mouse, asthma OVA aerosol airway model	Suppression of airway eosinophilia, Th2 cytokine synthesis, bronchial hyperreactivity, Treg induction >10-fold anti-OVA IgA increment in lung (OVA + CTB *vs.* OVA only)	[49]
OVA myelinoligodendrocyteglycoprotein peptide (MOG 35–55)	conjugated	BALB/c, C57BL/6, induced EAE *ex* *vivo* incubation of B cells with Ag + CTB and *in* *vivo* transfer	IL-10 production by B cells; >50-fold increase of anti-OVA Foxp3 + Treg; prevention of EAE induction	[50]
OVA	C-terminal, DNA vaccine(pSV-OVA-CTB) combined with recombinant Tiantan vaccinia (rTTV-OVA-CTB)	C57BL/6 mouse, oral, i.n. prime with pSV-OVA-CTB, i.m. boost with rTTV-OVA-CTB	Triplication of Ag-specific T cells compared to same DNA vaccines lacking CTB	[51]
HIVrgp160	unfused, combined with proteasomes or emulsomes	BALB/c mouse, oral/i.n.	Serum IgA, 20-fold increase (proteasome *vs.* proteasome + CTB), intestinal IgA, 60-fold increase (proteasome *vs.* proteasome + CTB)	[52]
HIV gp120 coding DNA (DNA-EnvB) + MVA-EnvB (boost)	unfused, +/− IL-12 coding DNA	BALB/c mouse, i.n.	Ag-specific T cell response duplicated (DNA-EnvB + IL-12 + CTB *vs.* DNA-EnvB)	[53]
Envelope GP1455m HIV	unfused	BALB/c mouse, i.m.	IgG titers 10-fold increased (DNA + CTB *vs*. DNA only)	[54]
HIV membrane proximal (ectodomain) region of gp41	C-terminal	BALB/c mouse. i.n., liposome conjugated + CT, i.p. boost without CT	Serum IgG and vaginal IgA detected, titer increased 100-fold, however, not all mice responding	[31]
Hemagglutinin fused to major immunodominant region (MIR) of Hepatitis B virus coreprotein (HBc)	unfused, mixed with 0.2%CT	BALB/c mouse, *in* *vivo* challenge withInfluenza, i.n.	50% increase of survival at low Ag dose (HA + CTB *vs*. HA)	[55]
Adhesin fromStreptococcus (AgI/II)	fused to CTA2, CTBCoexpressed but unfused	BALB/c mouse, i.n. or orally, +/− CT, −/+ Al(OH)_3_, no comparison to AgI/II only	IgG and IgA titers analyzed CT and AL(OH)_3_ increase IgG and IgA titers 2–10 fold when added to fusion construct CT augments serum IgA titers over 2-fold compared to Al(OH)_3_	[23]
Fimbria2 (*Bortedella pertussis*)	C-terminal	BALB/c mouse respiratory model, *Bortedella* *pertussis* i.p. or i.n.	IgG in serum close to ducplicated after intranasal vaccination, IgG in bronchoalveolar fluid 4-fold increased (CTB-Fim2 *vs.* recombinant Fim2)	[22]
Urease fragments, 5T and B cell epitopes (*Heliobacter* *py lori*)	C-terminal, transformed *Lactococcus* *lactis*	BALB/c, oral immunization with live Lactococcus	>100-fold reduction of gastric colonization by *H.* *pylori*	[56]
CTB	N/A	Monocyte-derived DC	CTB interfers with LPS-induced maturation IL-12 production reduced by >50% CTB-treated DC reduce IFN-γ by Tcells (>60%), IL-10 and TGF-β unchanged	[57]
CTB	N/A	suckling mouse model, femalesimmunized i.p., s.c. or i.n., pupschallenged with *V.* *cholerae*	60%–100% survival of offsprings from immunized *vs*. PBS treated females	[58]
CTB	unfused, Asn to Glnsubstitution at 4thglycosylation position	Macaque, oral BALB/c mouse, oral	Mice: protection against diarrhea, detection of serum IgG, fecal IgA; Macaques: detection of serum IgG, no fecal IgA	[36]
Naf1 *(Naegleria* *fowleri*)	unfused	BALB/c mouse, amoebic meningoencephalitis, i.n.	60% *vs*. 0% Survival (Naf1 + CTB *vs.* Naf1) of miceinfected with N.f.	[59]
Plasmodium vivaxookinete surfaceprotein (P *vs*. 25)	conjugated or mixed withCTB	BALB/c mouse, i.n. or i.p., +/− IFA	10-fold increase of IgG sera titers using conjugated protein *vs*. P *vs.* 25 only or P *vs.* 25 + CTB	[60]
*Plasmodium yoelii* Merozoite surface protein-1(MSP1) C-terminal region	conjugated to CTB or mixed with CT or IFA	C57BL/6 mouse, i.n., s.c., immunized mice challenged with lethal dose of *P.* *yoelii*	>10-fold increase of IgG sera titer (conjugated *vs*. MSP1 only), conjugated MSP + CT or IFA leads to further 5–10 fold titer increase compared to conjugated only, full protection against parasite only when mixing conjugated MSP with CT or IFA (survival 10/10 *vs*. 0–2/10)	[20]
Human Pro-insulin	C-terminal	monocyte-derived dendritic cells(MoDCs), human, *in* *vitro*	Tolerogenic effect on DCs, indoleamine 2,3-dioxygenase upregulation	[61]
Insulin	C-terminal +/− GFP	NOD mouse, T1D model, oral feeding	50% reduction of T1D incidence	[29]
MBP	C-terminal	3× TgAD mice (Alzheimer disease), oral	Reduction of amyloid loads by 70% in hippocampus and cortex brain region	[35]
Human GAD55	C-terminal, recombinant vaccinia virus	NOD mouse, i.p., +/− CFA	Diabetes reduction between 50 and 20% compared to same vaccine w/o CFA	[62]
amyloid-β peptide (Aβ42)	C-terminal	B6C3-Tg (APPswe, PSEN1dE9)transgenic mice (Alzheimer), oral	Ab titers augmented but n.s. (Aβ42 *vs.* CTB-Aβ42) Learning improved but n.s. (Aβ42 *vs.* CTB-Aβ42)	[28]

MVA: modified vaccinia Ancara; gp: glycoprotein; IFA: incomplete Freund’s adjuvant; CT: cholera toxin; i.n.: intranasal; i.p.: interperitoneal; s.c.: subcutaneous; MBP: myelin basic protein; GAD: glutamic acid decarboxylase; CFA: complete Freund’s adjuvant; OVA: ovalbumin.

CTB may also be introduced into DNA vaccines. For example, it has been shown that CTB fused to ovalbumin formulated as DNA vaccine leads to a strong anti-OVA T cell response when combined with a recombinant vaccinia boost regimen [51], and salivary IgA levels directed against the *Streptococcus mutans* wall associated protein A increased when a CTB encoding plasmid was coinjected with the protein A coding plasmid [63]. CTB may also be coadministered together with DNA vaccines that do not code themselves for CTB [54]. CTB activates murine bone marrow derived dendritic cells and macrophages cells via Toll-like receptor signaling pathways, leading to the expression of chemokines, mainly Th2 (IL-4, IL-10) but also Th1 cytokines (IFN-γ), and inflammatory cytokines (IL-1β) *in vitro* and *in vivo* [53,59]. The generation of Th2 *versus* Th1 cytokines is independent from the route of immunization or the Ag as in several studies using one route and one Ag both types of cytokines were observed simultaneously [17,59]. When CTB and a DNA vaccine encoding for HIV Env were coinjected intramuscularly into mice, the response generated against Env was enhanced 10-fold, approximately, compared to the DNA vaccine administered alone [54]. It is possible, however, that some of these observations were due to contamination with LPS as a different report found that CTB can imprint dendritic cells to promote IgA production by B cells *in vitro* only in presence of LPS [64]. Direct interaction between CTB and TLR4 has nevertheless been show via ELISA [65].

As mentioned above, CTB has been used as adjuvant without being physically linked to the Ag. In the ovalbumin (OVA) asthma model, induced by aerosol formulation of the Ag and administration via the airway, CTB combined with OVA led to the suppression of airway eosinophilia, bronchial hyperreactivity and secretion of Th2 cytokines, *i.e.*, IL-4, IL-10 and IL-5 that were reduced 2–5 fold, while IFN-γ was two-fold upregulated. Ag-specific IgA concentration in the lung was more than 10-fold incremented in comparison to administration of OVA alone [49]. Unfused CTB has also been combined with HIV recombinant gp160 and proteosomes [52] where it led to a 20- and 60-fold increase of serum and intestinal IgA, respectively. Hemagglutinin, formulated as a fusion protein with the major immunodominant region of the hepatitis B virus core protein (HBc) increased the survival rate of BALB/c mice challenged with Influenza virus by 50% when administered together with instead of without CTB [55]. CBT as unfused adjuvant was further used successfully in prevention infection against amoeba *Naegleria fowleri* (see below). Nevertheless, coadministration of CTB with the Ag without coupling the proteins is not always successful. In a detailed study the authors compared the immunogenic effect of the *Plasmodium vivax* ookinete surface protein Pvs25, injected subcutaneously either alone, coinjected with CTB, or coupled chemically to CTB, with or without incomplete Freund’s adjuvant (IFA), or with or without CT as additional adjuvant. Ab titers were indistinguishable when either Ag alone or Ag mixed with CTB was injected. Coupling to CTB augmented titers 16-fold. Ab titers of free Ag plus IFA *versus* free Ag incremented 19-fold, thus IFA and CTB were approximately comparable in terms of adjuvant efficacy. When IFA was combined with CTB-conjugated Ag, the titers incremented another 10-fold. The addition of CT to these three Ag formulations was analyzed via intranasal immunization. CT had a considerable effect (>10-fold increase of Ab titers) when combined with CTB-conjugated Ag, but no effect when combined with Ag alone or Ag plus CTB [60]. This study shows an advantage of conjugated Ag-CTB formulations, *i.e.*, the possibility to combine it with another adjuvant leading to its further potentiation. When OVA was either injected as mixture with CTB or conjugated to CTB, the conjugated version elicited a 10-fold stronger Ab response [48]. As mentioned above, CTB can lead to activation of APCs and to the secretion of chemokines, thereby enhancing immune responses. This could explain why CTB administered in an uncoupled form augments the immune response in some cases, although the latter two mentioned studies argue that coupling of CTB to the Ag is more efficient.

## 6. CTB as Vaccine Adjuvant in Infectious Diseases

As mentioned above, the presence of GM1 on the gut epithelium facilitates the entry of CTB into the body and the access to the immunological system. CTB has been used for two apparently contradictory goals, to improve protective immunity against infectious diseases and to reduce undesired autoimmune reactions.

As for protective immunity against infections, oral coadministration of CTB with recombinant Nfa1 protected mice against infection with the amoeba *Naegleria fowleri*, that can exist as a virulent pathogen causing amebic meningoencephalitis in humans and experimental animal models [59]. Nfa1 is a protein localized in the pseudopodia of the pathogen, and antibodies against this protein have been reported to reduce its pathogenicity [66]. In the study, IgG as well as IgA titers increased when CTB was added as adjuvant, leading to a 60% survival rate compared to 0% of animals immunized with Nfa1 only [59]. An enhanced Ab response to the HIV Env protein encoded by a DNA vaccine when coadministered with CTB was already mentioned above [54]. Similarly, an elevated CD8 response was observed in mice immunized with a DNA vaccine coding for HIV gp120 when coadministered nasally/mucosally together with CTB and plasmid-encoded IL-12 [53]. As mentioned above, CTB coupled to merozoite surface protein-1 (MSP1-19) enhances the protection of mice against *Plasmodium yoelii* infection, a murine malaria model system, when administered either intranasally or subcutaneously, leading to increased serum IgG titers [20]. In a murine gastritis model a multiple epitope vaccine (CTB-UE) directed against *Heliobacter pylori* reduced IL-17 in the plasma, which went in parallel with a reduction of the pathogen burden in the stomach [67]. Off note, CTB immunization, independently of the route of administration (intranasally, i.p. or subcutaneously), can lead to protection in the suckling offspring as has been shown in mice [58].

## 7. CTB in Immune Suppression

CTB has been analyzed for its capacity to induce immune suppression against allergic reactions and autoimmune disorders. Allergen epitopes have been successfully expressed as fusion proteins with CTB, such as Japanese Cedar pollen-derived T cell epitopes (Cry J 1 and Cry J 2 antigen) [25]. An experimental allergic rhinitis setup based on OVA as model antigen was used to analyze the effect and mechanism of CTB coadministration. CTB reduced several symptoms including TH2 cytokines, airway eosinophilia, and bronchial hyperreactivity. Mechanistically, CTB interfered with the capacity of dendritic cells to generate a TH2 response, increased their capacity to generate Foxp3+ Treg that, however, were not able to confer protection, and led to the production of IgA. Consequently, protection was obtained by the transfer of B cells from CTB treated animals [49]. A recent *in vitro* study using human dendritic cells (DC) has analyzed the mechanism further by which CTB in combination with autoantigen might contribute to active immune suppression. Co-culture of CTB fused to the autoantigen insulin with DCs, but neither insulin alone nor CTB alone induced up-regulation of the tryptophan catabolic enzyme indoleamine 2,3-dioxygenase (IDO1) that has been shown to have immunosuppressive function [61]. IDO1 expression was linked to NF-kB signaling as its blockage downregulated its production.

B cells have been shown to play an important role in CTB-mediated sublingual tolerance induction. In B cell deficient mice, the induction of Treg was reduced compared to wild type animals. This could be corrected by adoptive B cell transfer prior to tolerance induction. However, B cell-sufficient as well as B cell-deficient mice treated sublingually with CTB-OVA suppressed an anti-OVA response after parenteral challenge with the Ag. It was shown that in B cell deficient mice, Foxp3-CD4+ T cell expressing TGF-β increased strongly [68], leading the authors to speculate about two parallel pathways leading to oral tolerance induction, one being dependent on B cells but not the other. B cell-dependent oral tolerance was further found to necessitate expression of FcγRIII by these cells [69]. An alternative approach implicating B cells to induce tolerance has been their *in vitro* incubation with CTB-coupled Ag and their subsequent transfer to mice. This led to TGF-β and IL-10 expression by these cells *in vivo* and subsequently to the generation Foxp3+ regulatory cells. This was tested successfully using either OVA as model antigen or myelin oligodendrocyte glycoprotein peptide 35–55, the latter of which could be shown to prevent experimental autoimmune encephalomyelitis in this format [50].

In type 1 diabetes, CTB has been used as adjuvant to prevent diabetes in a relevant mouse model, the autoimmunity prone non-obese diabetic (NOD) mouse. Most of these reports, using CTB as a fusion protein, in a conjugated or unconjugated form or included in recombinant vaccinia viruses, in combination with a variety of autoantigens relevant to this disease such as insulin or glutamic acid decarboxylase, have advertised success in disease prevention [70,71], claiming the induction of regulatory T cells or a Th2 response. However, we recently came to a different conclusion by employing a MHC-tetramer CD4 T cell tracing approach in NOD mice. Using disease-relevant peptides fused to CTB and administrated orally we showed that in NOD mice Ag-specific CD4 T cells were not tolerized, but rather activated after parenteral challenge with the peptides, despite the generation of Ag-specific Foxp3+ CD4 T cells. Likewise, diabetes was not prevented in our hands. However, in diabetes-resistant F1 mice generated by crossing diabetes prone NOD mice with disease-resistant C57BL/6 mice, oral tolerance, *i.e.*, the inability of Ag-specific T cells to proliferate *in vivo* after parenteral immunization, was restored using the above-mentioned prime-boost regimen [17]. These results show that when analyzing oral tolerance induction by CTB to treat autoimmune diseases, a background truly prone to autoimmunity such as the NOD mouse is needed in order to gain meaningful insights. This is easier to achieve in the case of type 1 diabetes than in diseases currently needing induced model systems such as EAE.

Since CTB can prevent infection but also autoimmune reactions, the question is how these two apparently opposite immune reactions can be achieved by the same adjuvant. As we have mentioned, CTB can induce Th1 and Th2 cytokines, thus leading to the generation of neutralizing Abs helpful in the protection against infectious diseases and activating protective T cell responses. When using CTB to prevent autoimmune diseases, the balance between Th1 and Th2 cytokines will likely decide whether the response will be protective rather than deleterious. Since in this scenario autoantigens are employed, this seems to generally favor Th2 responses and regulatory T cell generation or activation.

## 8. Conclusions

The lack of toxicity combined with the stability and relative ease to express CTB either by its own or fused to peptides or proteins has made CTB an adjuvant easy to handle. The possibility to express the protein in a large variety of organisms further broadens its application potential. Its capacity to reduce antigen amounts up to 100-fold for immunization, due to its efficient binding to APCs and epithelium surfaces should make CTB a cost-effective adjuvant. To what extent it will serve to indeed prevent or reverse autoimmune diseases in humans remains an open question and a further challenge. However, our growing understanding of the immune system is likely to pave further roads in the future in order to modulate immune responses in the desired direction, be it immunostimulatory or immunomodulatory. CTB is currently being used in vaccines such as Dukoral, an anti-*V. cholerae* vaccine consisting of killed *V. cholerae* and recombinant CTB. CTB as adjuvant for human vaccines has been approved in Europe and Canada. Given the superb adjuvant effect of CTB it is likely that this protein should play an important role in the formulation of vaccines in the future.

## Abbreviations

CT: cholera toxin
CTB: cholera toxin subunit B
CTA: cholera toxin subunit A
GM1: monosialotetrahexosylganglioside
